# *Sasa veitchii* extracts suppress acetaminophen-induced hepatotoxicity in mice

**DOI:** 10.1186/s12199-017-0662-3

**Published:** 2017-06-12

**Authors:** Hiroki Yoshioka, Haruki Usuda, Hirohisa Fujii, Tsunemasa Nonogaki

**Affiliations:** 1 College of Pharmacy, Kinjo Gakuin University, 2-1723 Omori, Moriyamaku, Nagoya, Aichi 463-8521 Japan; 2 Department of Pharmacology, Shimane University Faculty of Medicine, 89-1 Enya-cho, Izumo, Shimane 693-8501 Japan

**Keywords:** ■■■

## Abstract

**Background:**

The aim of this study was to investigate the therapeutic effects of a *Sasa veitchii* leaf extract (SE) on acetaminophen (APAP)-induced hepatotoxicity.

**Methods:**

Seven-week-old male ddY mice were orally administered SE or saline (0.2 mL) once a day for a week. Twenty-four hours after the last pretreatment, the mice were intraperitoneally injected with 550 mg/kg APAP or saline under fasting conditions. The mice from each group were euthanized and bled for plasma analysis 2, 6, 24, and 72 h after the injection.

**Results:**

We found that pretreatment with SE significantly decreased hepatic injury markers (i.e., alanine aminotransferase and aspartate aminotransferase), oxidative stress (malondialdehyde and glutathione level), inflammatory cytokines, histological damage, c-jun N-terminal kinase activation, and receptor-interacting protein-1 activation. Further, SE pretreatment decreased Cyp2e1 expression and increased total antioxidant capacity in the liver.

**Conclusion:**

Our findings demonstrate that prophylactic SE treatment protects mice from APAP-induced hepatotoxicity through modulation of Cyp2e1 expression and antioxidant capacity.

## Background

Bamboo leaves have been extensively used in folk medicine as an antifebrile and antihypertensive medication for centuries [1, 2]. In addition, in Japan, due to their antimicrobial activity, bamboo leaves have been used to wrap sushi sheets to protect against bacterial spoilage. Recently, extracts of bamboo leaves were reported to have multiple biological activities including antioxidant activity and cancer prevention [3, 4]. Furthermore, previous studies have demonstrated the antitumor [5], antioxidant [1, 6], antiviral [7], anti-inflammatory [8], and anti-allergic [9] activities of the extract.

The liver is most vulnerable to attack by chemical toxic agents since it is one of the most internal organs with multiple functions such as detoxification and protein synthesis [10, 11]. Therefore, liver diseases are among the most serious health problems worldwide. Our previous investigation found that *Sasa veitchii* leaf extract (SE) prevented carbon tetrachloride (CCl_4_)-induced hepatotoxicity in mice [12], suggesting that SE might maintain liver homeostasis. Although CCl_4_ is commonly used in animal models to study chemical toxin-induced liver injury [13, 14], exposure to CCl_4_ does not reflect a real-life scenario. The most common etiologies of liver damage in real life are acute viral hepatitis A and B, drug-related liver injury, and the alcohol-acetaminophen syndrome (AAS). In terms of drug-related liver injury, acetaminophen (APAP) is well known. In addition, the main cause of acute liver failure in industrialized countries is APAP overdose. APAP is recognized as a popular analgesic and antipyretic drug at therapeutic doses. However, APAP can cause severe liver injury in animals and humans through its side effects [15]. The main mechanism of APAP-induced hepatotoxicity has been studied in detail [16–19] and occurs in multiple steps. First, APAP is majority metabolized by Cyp2e1 and minority by Cyp1a2. Cyp2e1 is the main Cyp enzyme that bio-activates APAP at low doses [16, 20]. In contrast, at a high dose, Cyp1a2 was shown to contribute to the bio-activation and toxicity of APAP [21]. Cyp-produced *N*-acetyl-p-benzoquinone imine (NAPQI) depletes glutathione (GSH) and covalently binds to proteins. Loss of GSH then permits increased formation of reactive oxygen and nitrogen species. As a result, oxidative stress is increased, leading to alterations in calcium homeostasis and initiation of signal transduction responses causing mitochondrial permeability transition. Mitochondrial permeability transition causes additional oxidative stress and inhibits ATP synthesis. Insufficient ATP triggers cell necrosis. Peripheral to these essential events, a number of inflammatory mediators such as cytokines and chemokines are induced that can modify toxicity.

Currently, the best antidote against APAP-induced hepatic injury is *N*-acetylcysteine (NAC). NAC effectively minimizes APAP-induced toxicity when injected within a short time after APAP overdose [22]. However, clinical studies have revealed that injection of NAC often causes undesirable side effects [23]. Therefore, a need exists for compounds or chemicals that offer maximum protection against APAP-induced hepatotoxicity without NAC’s side effects. The search for new drugs and novel therapeutic intervention strategies increasingly includes testing plant extracts and other natural products. It is generally accepted that natural products have multiple activities including anticancer and antioxidant [24, 25]. Owing to the critical and urgent need to explore the therapeutic potential of natural products and other compounds for prevention and treatment of hepatotoxicity, we evaluated the protective effects of SE against APAP-induced hepatic injury.

## Methods

### Animal experimentation

Six-week-old male ddY mice were purchased from Japan SLC (Shizuoka, Japan). They were housed under standard conditions of controlled temperature (24 ± 1 °C), humidity (55 ± 5%), and light (12-h/12-h light/dark cycle) and provided food and water ad libitum. All experiments were approved by the Institutional Animal Care and Experimentation Committee of Kinjo Gakuin University (No. 129).

### Preparation of SE

SE was obtained from Sunchlon Co., Ltd. (Nagano, Japan) and was prepared using the method described previously [9, 12]. One milliliter of SE was obtained from 2.82 g of *S. veitchii* leaves according to the company’s data. The diluted SE solution contained minerals, carbohydrates, fat, protein, and silicic acid, as shown in Table 1.

**Table 1:** Component of SE

Component	mg/mL
Cu-chlorophyll	250.00
Carbohydrate	8.17
Fat	3.58
Protein	3.08
Ash content	0.83
Silicic acid	0.42
Water	940.80
Minerals	μg/mL
Na	2250.00
Fe	5.67
Zn	1.83
Cu	733.33
Mn	0.18
Ca	11.67
Mg	15.00
K	86.67
P	18.33

### Treatment protocol

Mice were pretreated with 0.2 mL of SE or saline as a control once a day for a week by oral gavage. Mice from each group were fasted for 16 h, and 550 mg/kg APAP dissolved in saline/polyethylene glycol emulsion or vehicle only (as a control) was i.p. injected at 10 mL/kg body weight. The mice were euthanized and bled to obtain plasma 2, 6, 24, or 72 h following the injection. The resulting plasma samples were stored at −80 °C. The livers were harvested and stored at −80 °C or fixed in 15% phosphate-buffered neutral formalin (pH 7.4).

### Plasma biochemical analysis

Plasma activities of alanine aminotransferase (ALT) and aspartate aminotransferase (AST) were measured using the Transaminase CII kit (Wako Pure Chemical, Osaka, Japan) according to the manufacturer’s instructions, as previously described [26]. Plasma levels of tumor necrosis factor (TNF)-α (eBioscience, San Diego, CA, USA) were determined using commercially available ELISA kits, according to the manufacturer’s instructions. For relative quantification, calibration curves were prepared using standard solutions.

### Histopathological findings

For histological analysis, a portion of the left liver lobe from each animal was perfused with 15% phosphate-buffered neutral formalin (pH 7.4), dehydrated, and embedded in paraffin. Embedded tissues were sectioned at 4 μm.

For immunohistochemistry, paraffin-embedded sections were deparaffinized and rehydrated in a grated ethanol series. After antigen retrieval by proteinase K (Wako Pure chemical) and quenching of endogenous peroxidase by hydrogen peroxide (Wako Pure chemical), sections were incubated with rat anti-TNFα monoclonal antibody (Biolegend, San Diego, CA, USA) as primary antibodies (1:250 dilution) at 4 °C for overnight. The color reaction was developed with peroxidase-conjugated anti-rat IgG (Sigma-Aldrich, St. Louis, MO, USA) as secondary antibody (1:750 dilution). In addition, sections were counterstained with hematoxylin.

For the detection of nuclear DNA strand breaks, paraffin-embedded sections were stained with the terminal dUTP nick-end labeling (TUNEL) technique using an in situ apoptosis detection kit (Takara Bio, Shiga, Japan) according to the manufacture’s protocols. Sections were counterstained with hematoxylin.

For hematoxylin and eosin (H&E) stain, we previously described [26].

### Measurement of malondialdehyde and GSH levels in the liver

Total malondialdehyde (MDA) levels and total antioxidant capacity in the liver were examined using a colorimetric thiobarbituric acid reactive substances microplate assay kit and a colorimetric total antioxidant power assay kit, respectively (Oxford Biochemical Research, Oxford, MI, USA), according to the manufacturer’s protocols, as previously described [27]. Hepatic GSH levels were measured using GSSG/GSH quantification kit (Dojindo Laboratories, Kumamoto, Japan) according to the manufacturer’s instructions and as previously described [28].

### Western blot analysis

0.1-g liver sections were homogenized with 900 μL ice-cold phosphate-buffered saline containing protease inhibitor (Nacalai Tesque, Kyoto, Japan) and centrifuged at 18,000*g* for 20 min at 4 °C. The resulting supernatant for each sample was collected and protein level determined using the BCA protein kit (Nacalai Tesque). Protein samples (40 μg) were subjected to sodium dodecyl sulfate–polyacrylamide electrophoresis on a 10% gel and transferred to a polyvinylidene difluoride membrane. Rabbit anti-c-jun N-terminal kinase (JNK) polyclonal antibody, rabbit anti-phospho-JNK monoclonal antibody (Cell Signaling Technology, Beverly, MA, USA), and rabbit anti-Cyp2e1 polyclonal antibody (Enzo Life Sciences, NY, USA) were used as primary antibodies (1:1000 dilution) for immunoblotting. A peroxidase-conjugated anti-rabbit IgG (Cell Signaling Technology) was used as secondary antibody (1:3000 dilution). Mouse anti-receptor-interacting protein (RIP)-1 monoclonal antibody (Santacruz, California, CA, USA) and mouse anti-β-actin monoclonal antibody (MBL, Aichi, Japan) were used as primary antibody (1:1000 dilution) for immunoblotting. A peroxidase-conjugated anti-mouse IgG (MBL) was used as secondary antibody (1:5000 dilution). The immunoreactive bands were visualized with the ECL system (BioRad, Hercules, CA, USA).

### Isolation of total RNA and qRT-PCR assay

Total RNA was extracted from 0.1-g liver sections using the ISOGEN II kit (Nippon Gene, Tokyo, Japan). qRT-PCR was performed with One-Step SYBR PrimeScript PLUS RT-PCR kit (Perfect Real Time) (Takara Bio) using an Applied Biosystems 7300 system (Applied Biosystems, Foster City, CA, USA). PCR conditions were as previously described [28]. Primer pairs are shown in Table 2. Relative expression of each messenger RNA (mRNA) was determined using the standard curve method. The amount of each target mRNA quantified was normalized against that of GAPDH-encoding mRNA.

**Table 2:** Oligonucleotide primer sequences and PCR conditions for real-time RT-PCR

Gene (accession no.)	Primer sequences		PCR product length (bp)
	Sequence (5′ to 3′)		
Cyp1a2	Forward	GAC ACC TCA CTG AAT GGC TTC	103
(NM_009993)	Reverse	ACA CAA AGG GGT CTT TCC ACT G	
Cyp2e1	Forward	CAT TCC TGT GTT CCA GGA GTA CAA G	91
(NM_021282)	Reverse	GAT ACT TAG GGA AAA CCT CCG CAC	
GAPDH	Forward	TGG TGA AGG TCG GTG TGA AC	98
(NM_008084)	Reverse	GTC GTT GAT GGC AAC AAT CTC C	

### Statistical analysis

Statistical significance of the differences between two groups was estimated using a two-tailed Student *t* test. Multiple comparisons were made by one-way analysis of variance with post hoc Tukey–Kramer’s test. All statistical analyses were performed using the SPSS 19.0 software (Chicago, IL, USA). Values of *p* < 0.05 were considered statistically significant.

## Results

### Effect of SE on APAP-induced acute toxicity as assessed through evaluation of hepatic function markers

**Fig. 1:**
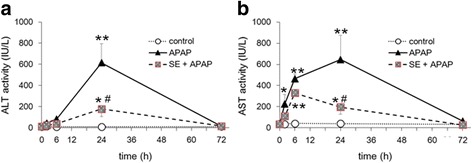
Effect of pretreatment with SE on levels of hepatic injury markers. Mice received SE or vehicle (saline) by oral gavage once daily for a week. Twenty-four hours after final SE administration, 550 mg/kg of APAP was injected intraperitoneally. Mice were fasted for 16 h before APAP injection. Plasma levels of hepatic injury markers were determined 2, 6, 24, and 72 h after administration of APAP. Panels **a** and **b** indicate ALT and AST, respectively. Data are plotted as mean ± SD for groups of six mice each. *Single asterisk* indicates *p < 0.05* versus control group, *double asterisk* indicates *p < 0.01* versus control group, and *number sign* indicates *p < 0.05* versus APAP group

In an initial animal experiment, we analyzed plasma ALT and AST activities (Fig. 1), which are markers of liver injury and dysfunction. Administration of APAP led to a slight increase in ALT activity at 2 and 6 h and a significant increase at 24 h (Fig. 1a). These levels decreased at 72 h. APAP increased AST activity beginning at 2 h, with the highest activity observed at 24 h (Fig. 1b). In contrast, pretreatment with SE attenuated APAP-induced ALT and AST activities at 24 h.

### Changes in hepatic MDA and GSH levels in APAP-exposed mice pretreated with SE

**Fig. 2:**
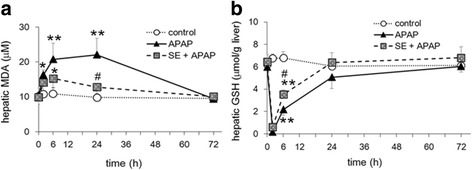
Pretreatment with SE counteracts the effects of acute APAP-induced toxicity on MDA and GSH levels. Animals treated as described in Fig. 1 were euthanized 2, 6, 24, and 72 h post-intraperitoneal injection, and the livers were harvested at necropsy. Liver specimens were assessed for MDA levels (**a**) and GSH levels (**b**). Data are plotted as mean ± SD for groups of six mice each. *Single asterisk* indicates *p < 0.05* versus control group, *double asterisk* indicates *p < 0.01* versus control group, and *number sign* indicates *p < 0.05* versus APAP group

Along with the measurement of liver function markers, we determined hepatic MDA level as a marker of lipid peroxidation. APAP treatment significantly increased MDA levels at 2, 6, and 24 h, as expected, and pretreatment with SE attenuated APAP-induced MDA upregulation at 24 h (Fig. 2a). We also measured hepatic GSH content, since APAP is well known to deplete hepatic GSH [16]. APAP exposure completely depleted hepatic GSH at 2 h and slightly recovered at 6 h. In addition, the GSH levels recovered beginning at 24 h (Fig. 2b). Although pretreatment with SE significantly decreased GSH levels compared to those observed for control, the depletion rate was significantly lower than that of the APAP group at all time we tested.

### Influence of SE on APAP-induced acute toxicity as assessed through evaluation of plasma TNFα levels

**Fig. 3:**
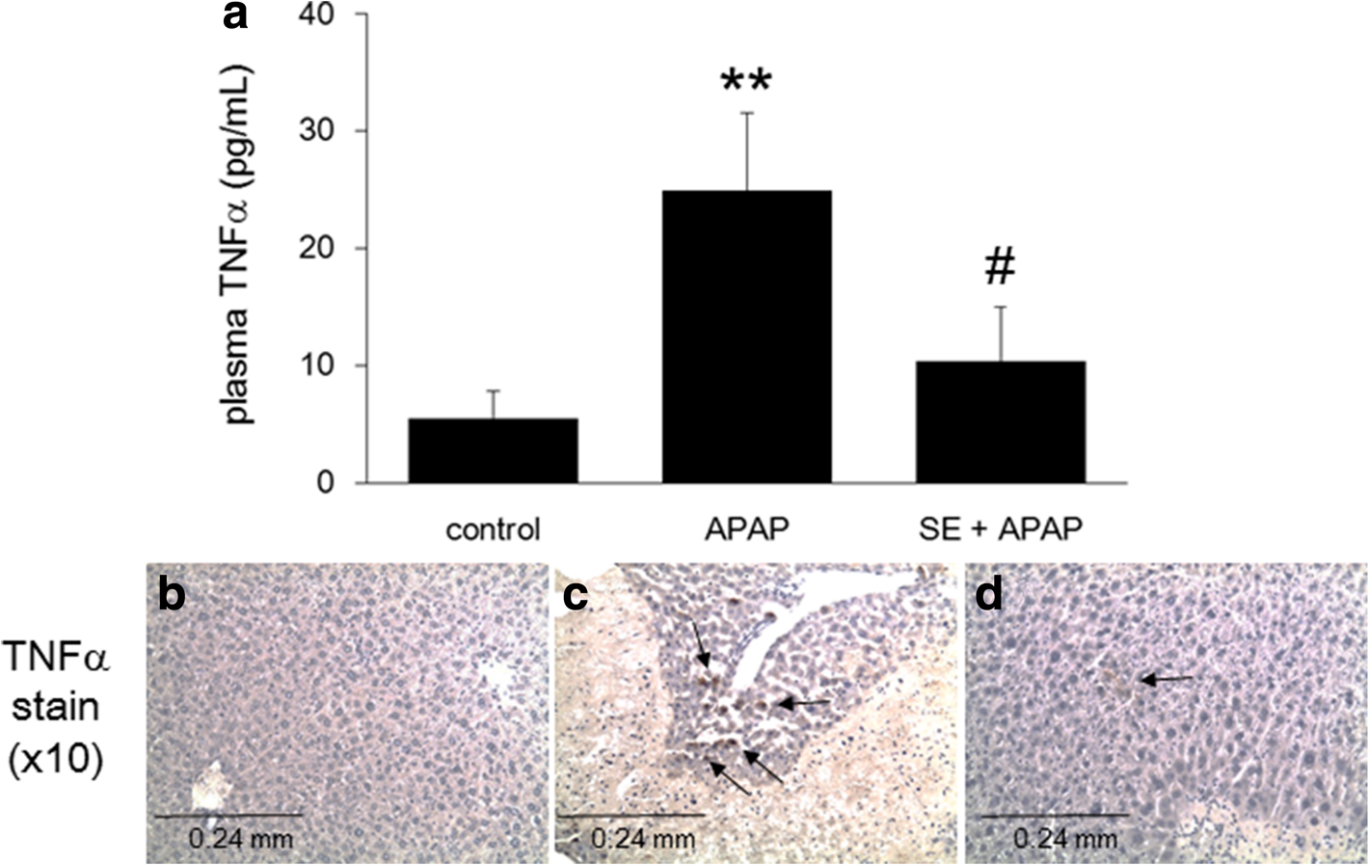
Pretreatment with SE counteracts the inflammatory response induced by acute APAP-induced toxicity. Animals treated as in Fig. 1 were euthanized at 24 h post-intraperitoneal injection, and plasma TNFα levels (**a**) were determined. Data are plotted as mean ± SD of groups of six mice each. *Double asterisk* indicates *p <0.01* versus control group, and *number sign* indicates *p <0.05* versus APAP group. Liver specimens were fixed and processed, and sections were stained with TNFα antibody. These micrographs provide ×10 magnified images of representative TNFα-stained sections of the livers obtained from control (**b**), APAP (**c**), and SE + APAP (**d**) group animals. *Black arrows* indicate TNFα production

Exposure to APAP is known to elevate inflammatory responses [19]. In the present study, we measured plasma TNFα as a representative inflammatory cytokine (Fig. 3a). Our results showed that APAP treatment significantly upregulated hepatic plasma TNFα levels, which were significantly decreased by pretreatment with SE. Moreover, in parallel with plasma TNFα, we also investigated hepatic TNFα production by immunostaining since the liver is the main target of APAP-induced toxicity. We observed hepatic TNFα-production by APAP exposure (Fig. 3c). In addition, pretreatment with SE prevented APAP-induced inflammatory response in the liver (Fig. 3d).

### Effect of SE against APAP-induced acute toxicity as assessed through evaluation of hepatic structure

**Fig. 4:**
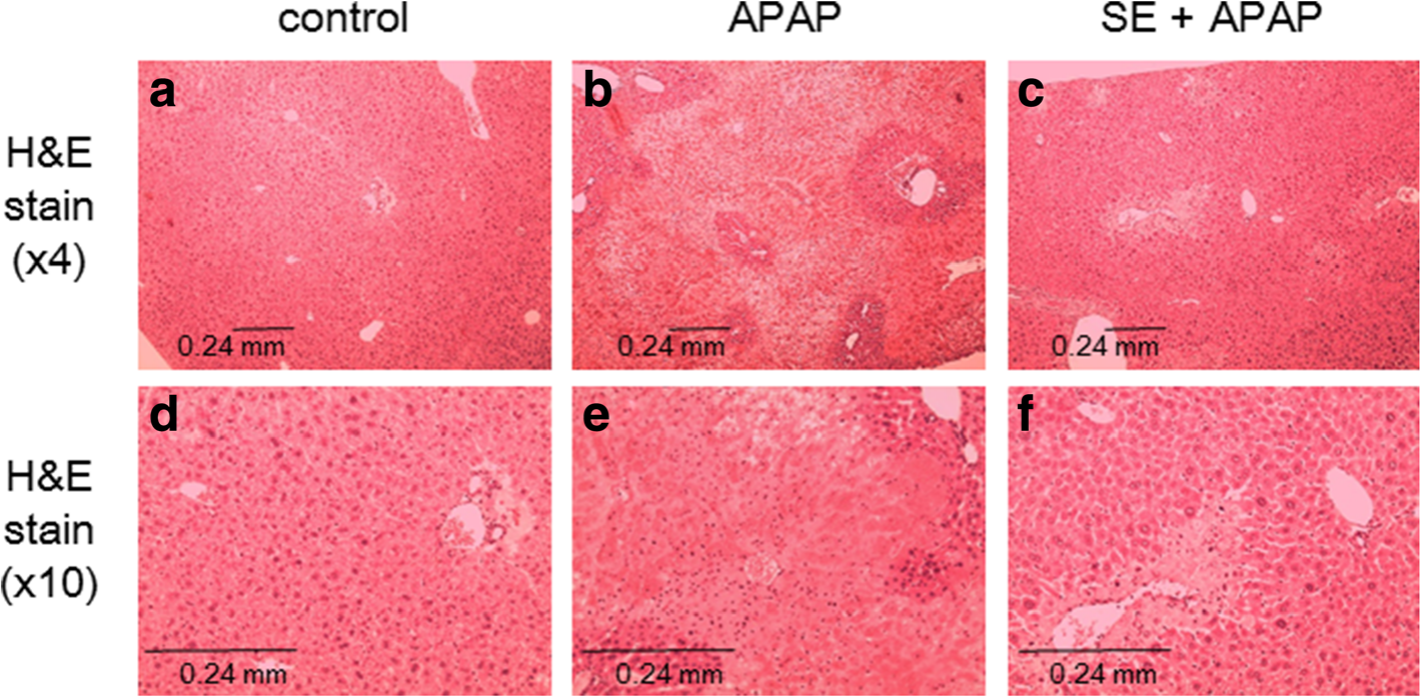
SE pretreatment protects animals from acute APAP-induced hepatotoxicity, as assessed by H&E staining. Animals treated as in Fig. 1 were euthanized at 24 h post-intraperitoneal injection, and the livers were harvested at necropsy. Liver specimens were fixed and processed by standard methods, and sections were stained with H&E. These micrographs provide ×4 (**a**, **b**, **c**) and ×10 (**d**, **e**, **f**) magnified images of representative H&E-stained sections of the livers obtained from control (**a**, **d**), APAP (**b**, **e**), and SE + APAP (**c**, **f**) group animals. The image in **b** and **e** reveals severe necrosis and apoptosis in an APAP-exposed animal. In contrast, although necrosis is observed around the central vein in the SE + APAP group, the necrosis area is lower than that in the APAP-exposed group

**Fig. 5:**
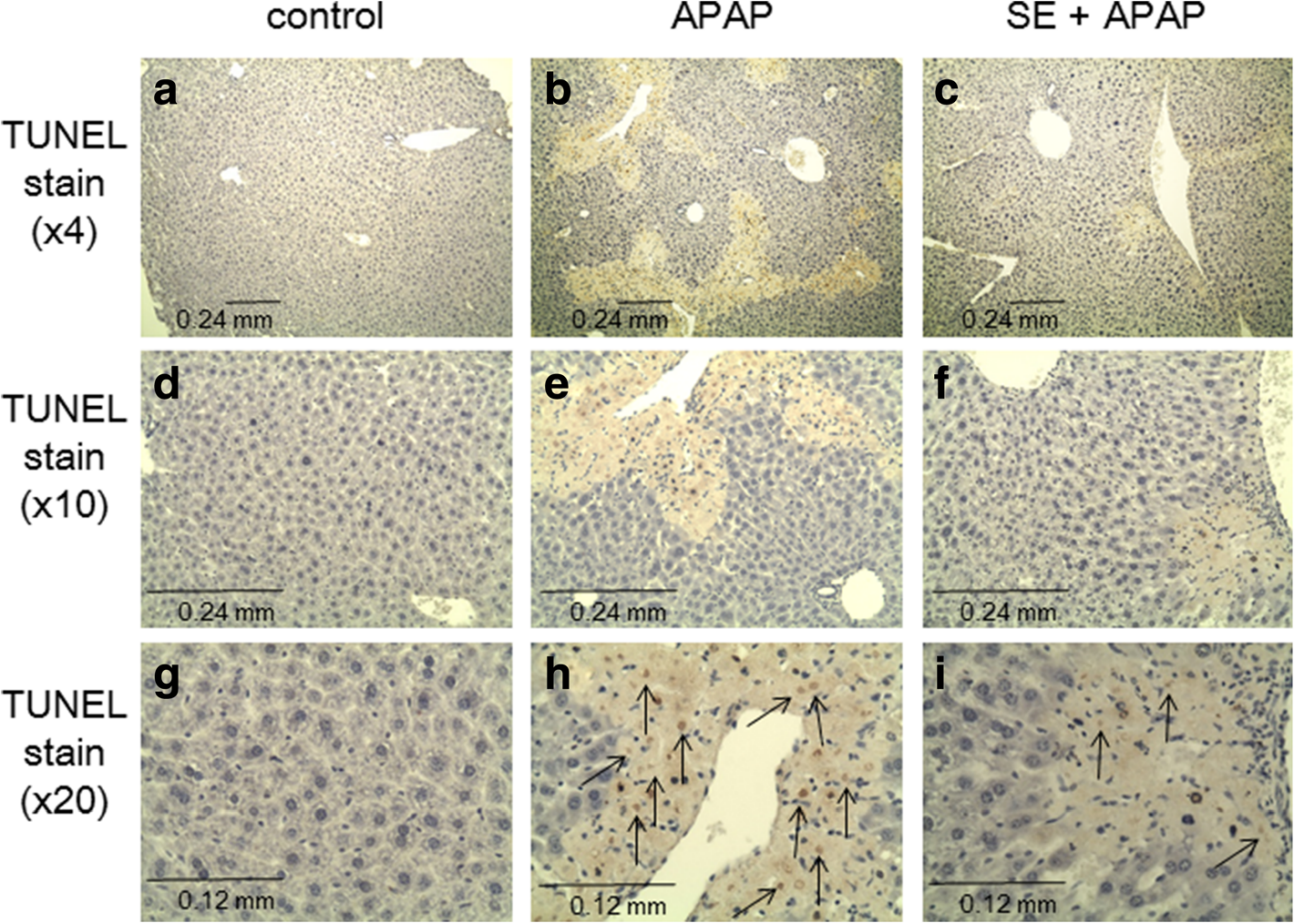
SE pretreatment protects animals from acute APAP-induced hepatotoxicity, as assessed by TUNEL staining. Animals treated as in Fig. 1 were euthanized at 24 h post-intraperitoneal injection, and the livers were harvested at necropsy. Liver specimens were fixed and processed, and sections were stained with TUNEL. These micrographs provide ×4 (**a**, **b**, **c**), ×10 (**d**, **e**, **f**), and ×20 (**g**, **h**, **i**) magnified images of representative TUNEL-stained sections of the livers obtained from control (**a**, **d**, **g**), APAP (**b**, **e**, **h**), and SE + APAP (**c**, **f**, **i**) group animals. Black arrows indicate nuclear DNA strand breaks

To further investigate the protective effect of SE against APAP-induced hepatotoxicity, we conducted histopathological studies (Fig. 4). Liver sections stained with H&E showed normal cell morphology, well-preserved cytoplasm, and a clear, plump nucleus in the control groups (Fig. 4a, d). However, in APAP-injected mice, severe necrosis and apoptosis were observed (Fig. 4b, e). Although mice pretreated with SE showed necrosis around the central vein (CV), the necrotic area was lower than that in the APAP-exposed group (Fig. 4c, f). Moreover, we investigated the protective effect of SE on nuclear DNA fragmentation and DNA strand breaks (TUNEL assay, Fig. 5). The TUNEL assay showed extensive DNA damage in cells around CV in APAP-exposed animals (Fig. 5b, e). In addition, nuclear DNA fragment was also observed (Fig. 5h). In contrast, pretreatment with SE attenuated nuclear DNA fragmentation and DNA strand breaks, respectively (Fig. 5c, f, i).

### Protective effect of SE through JNK and RIP inactivation

**Fig. 6:**
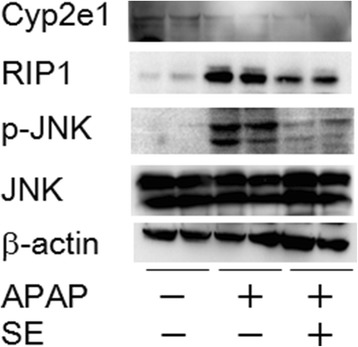
Protective effect of SE through JNK and RIP inactivation. Animals treated as in Fig. 1 were euthanized at 24 h post-intraperitoneal injection. The livers were harvested, and proteins were collected. Data indicate hepatic Cyp2e1, RIP1, phosphorylated JNK, and JNK activation, respectively. As internal control, we measured β-actin

According to several reports, JNK activation plays a key role in APAP-induced hepatic injury [29–31]. Moreover, APAP-induced hepatic injury is thought to involve primarily hepatocyte necrosis, and RIP1 is emerging as a key regulator of necrotic cell death [32, 33]. We examined the protective effect of SE on JNK and RIP1 activation in APAP-exposed mouse (Fig. 6). As expected, APAP significantly increased the level of hepatic phosphorylated JNK and RIP1 activation. In addition, APAP-induced hepatic JNK phosphorylation and RIP1 activation were attenuated by pretreatment with SE. Moreover, we observed hepatic Cyp2e1 level. Although APAP group and SE + APAP groups indicated a tendency of Cyp2e1 decrease, these two groups in Cyp2e1 level were comparable.

### Protective effect of SE through evaluation of Cyp induction and antioxidant capacity

**Fig. 7:**
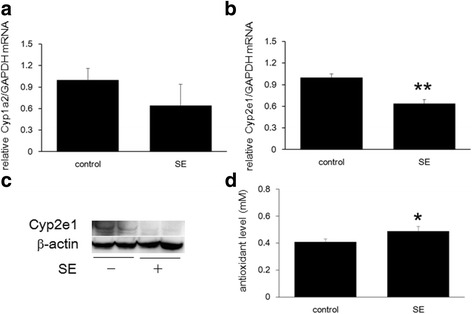
Cyp induction and antioxidant effect of SE. Mice received SE or vehicle (saline) by oral gavage once daily for a week. Twenty-four hours after final pretreatment, the livers were harvested at necropsy. Panels **a** and **b** indicate *Cyp1a2* and *Cyp2e1* mRNA levels, respectively. Panel **d** indicates hepatic antioxidant capacity. Data are plotted as mean ± SD of groups of six mice each. *Single asterisk* indicates *p < 0.05*, and *double asterisk* indicates *p < 0.01* versus control group. Data in **c** indicate hepatic Cyp2e1 protein levels. As internal control, we measured β-actin

To further investigate the protective effect of SE against APAP-induced hepatotoxicity, we measured hepatic *Cyp1a2* and *Cyp2e1 mRNA* expression (Fig. 7a, b). SE treatment decreased *Cyp1a2* expression and significantly decreased *Cyp2e1* mRNA. In addition, the same tendency was also observed in Cyp2e1 protein levels (Fig. 7c). These data suggest that SE modulates Cyp expression.

We also studied the effect of SE on hepatic antioxidant capacity (Fig. 7d). Total antioxidant capacity was significantly increased by SE treatment, indicating that SE has significant antioxidant activity.

## Discussion

This study demonstrated that pretreatment with SE can prevent APAP overdose-induced acute toxicity, as assessed by evaluating blood function markers, oxidative stress (MDA and GSH level), inflammatory responses, JNK activation, and RIP1 activation in the liver. In addition, SE also prevented APAP-induced necrotic and apoptotic cell death, respectively. Although apoptosis occurred by APAP treatment, ratio of TUNEL-positive cells was lower compared with necrosis area. Moreover, TUNEL-positive nucleus is observed within the area of necrosis. That suggests TUNEL-positive cells in APAP-induced cell death reflect necrosis-mediated DNA fragmentation not apoptotic cell death because caspase-3, that is well known to apoptosis marker, was not elevated by APAP treatment [34, 35].

In the present study, although we cannot show whether TUNEL-positive cells were necrosis-mediated DNA fragmentation or apoptotic cell death, these are consistent with past literature that APAP-induced liver injury results from mainly necrotic rather than apoptotic cell death [34, 36].

Since APAP-induced hepatotoxicity occurs in multiple steps, SE may have protective effects against several steps. Initially, we hypothesized that SE acts as an antioxidant. Oxidative stress is a mechanism that has been postulated to be important in the development of APAP-induced hepatotoxicity [16, 37, 38]. Numerous studies have reported that antioxidants can prevent hepatic damage by counteracting free radicals and preventing lipid peroxidation [39, 40]. We found that pretreatment of APAP-treated mice with SE reduced levels of hepatic MDA, a naturally occurring product of lipid peroxidation. Several literature reports have hypothesized that SE exhibits antioxidant activity because many new flavonoids and polyphenols have been discovered in *S. veitchii* [41, 42]. Our hypothesis is supported by the well-known antioxidant properties of such compounds [43, 44], as well as our observation of SE-induced increase in hepatic antioxidant capacity. Chlorophyll might also be a candidate active molecule since it has demonstrated antioxidant activity and is present in abundance (250 mg/mL). Further, Serpeloni et al. [45] have demonstrated that 13-day oral treatment with chlorophyll (0.5 mg/kg) suppressed cisplatin-induced oxidative stress in mice. Although the period of SE administration was shorter in our study, the chlorophyll content in our SE was much greater than 0.5 mg per day. Therefore, the chlorophyll in SE could have contributed to the suppression of APAP-induced oxidative stress in our study.

In clinical practice, NAC has been used to treat patients with APAP overdose since it has the potential to promote hepatic GSH synthesis [46] and APAP is well known to deplete GSH [47]. Moreover, GSH injection is effective in mice. However, GSH upregulation is not observed upon pretreatment with SE. Taken together, our data indicate that SE may protect against APAP toxicity by ameliorating the impairment of antioxidant systems except for GSH.

Another possible explanation for our results is that the protective effect of SE against APAP-induced toxicity may possibly reflect inhibition of some CYPs, which would in turn prevent the formation of toxic NAPQI. Cyp2e1 gene knockout mouse or Cyp2e1 inhibition by an inhibitor such as disulfiram or natural product such as resveratrol and schisandra attenuated APAP-induced hepatotoxicity in mice [48–50]. In addition, Cyp1a2 knockout mice showed reduced APAP-induced toxicity [51, 52]. We used qRT-PCR to investigate the effect of SE on *Cyp2e1* and *Cyp1a2* mRNA expression and showed that SE significantly decreased *Cyp2e1* gene expression. Furthermore, the same tendency was also observed in Cyp2e1 protein levels. These might provide an additional explanation of the protective mechanism of SE against APAP toxicity. In addition, we observed hepatic Cyp2e1 level 24 h after APAP treatment. Although APAP group and SE + APAP groups indicated a tendency of Cyp2e1 decrease, these two groups in Cyp2e1 level were comparable. Several literatures reported Cyp2e1 was degraded after administration of toxic doses of APAP, possibly through interaction between Cyp2e1 and NAPQI or other metabolites produced from Cyps-mediated metabolism of APAP. Our data was consistent with these reports [53, 54]. On the other hand, in SE + APAP group, we cannot prove Cyp2e1 decrease was whether depending on APAP-induce degradation or inhibition potential by SE. Further investigation is needed to elucidate this question in the future research.

An additional explanation is that SE protects against APAP-induced hepatotoxicity via its anti-inflammatory potential. It is well known that drug toxicity such as that due to APAP causes hepatocyte necrosis, which results in neutrophil and monocyte infiltration. It is reported that Kupffer cells activate the expression of pro-inflammatory cytokines such as TNFα and IL-6 [55, 56]. Our study also showed that APAP administration significantly induced TNFα, which was inhibited by SE pretreatment. Nuclear factor (NF)-κB is an important transcription factor for inducible inflammatory cytokine expression [57, 58]. In vitro assays from other groups have shown that SE inhibited NF-κB activity [8], suggesting that our explanation is plausible. However, in the current study, we cannot elucidate whether repression of inflammatory response is due to direct effect or upper event such as Cyp inhibition and ROS decrease since inflammatory response is latter event in APAP-induced hepatotoxicity. Hence, further investigation might be needed to prove whether SE’s anti-inflammatory potential contributes to protection against APAP-induced hepatotoxicity or not.

## Conclusion

In conclusion, we demonstrated that pretreatment with SE suppresses APAP-induced hepatic injury and hypothesize that the hepatoprotective mechanisms of SE are wide-ranging, including inhibition of Cyp2e1 expression and antioxidant activity. Although further investigation is needed to clarify the detailed protective mechanisms and main component(s) of SE, these findings are expected to contribute to self-medication options against acute hepatic injury and disease.
